# Increasing age is associated with elevated circulating interleukin-6 and enhanced temporal summation of mechanical pain in people living with HIV and chronic pain

**DOI:** 10.1097/PR9.0000000000000859

**Published:** 2020-10-22

**Authors:** Deepika E. Slawek, Jessica S. Merlin, Michael A. Owens, Dustin M. Long, Cesar E. Gonzalez, Dyan M. White, Salvador A. Lopez, Sonya L. Heath, Burel R. Goodin

**Affiliations:** a Department of Medicine, Albert Einstein College of Medicine, Bronx, NY, USA; b Department of Medicine, Montefiore Medical Center, Bronx, NY, USA; c Divisions of General Internal Medicine and Infectious Diseases, University of Pittsburgh, Pittsburgh, PA, USA; Departments of d Psychology and; e Biostatistics, University of Alabama at Birmingham, Birmingham, AL, USA.; f Department of Medicine, Division of Infectious Diseases, University of Alabama at Birmingham, Birmingham, AL, USA

**Keywords:** HIV, Aging, Pain, Inflammation, Modulation

## Abstract

**Introduction:**

In the current antiretroviral treatment era, people living with HIV (PLWH) can achieve a near-normal life expectancy. However, as PLWH grow older, they are increasingly prone to developing chronic health conditions including pain. One possible explanation for increased pain in older populations with HIV is that aging is associated with inflammation and altered pain modulatory processes (ie, increased facilitation and decreased inhibition).

**Objectives:**

Our study aimed to examine how age affects endogenous pain modulatory processes in PLWH with chronic pain and to examine how age affects serum inflammatory cytokines.

**Methods:**

A total of 80 PLWH (median CD4^+^ = 646; 24% detectable viral load >50; 99% on antiretroviral therapy) with chronic pain provided demographic information (age, sex, and race) and completed standardized questionnaires to assess mood and clinical pain severity. Blood assays were completed to determine circulating levels of interleukin-6 (IL-6) and tumor necrosis factor-α, as well as CD4^+^ and HIV viral load. Temporal summation of mechanical pain and conditioned pain modulation assessed pain facilitation and inhibition, respectively.

**Results:**

Mean age was 48.9 (8.2) years; range: 26 to 67, with 45% >50 years. In adjusted multiple regression models, increasing age was associated with elevated levels of circulating IL-6 (*P* < 0.01), but not tumor necrosis factor-α. Increasing age was also associated with enhanced temporal summation of mechanical pain (*P* < 0.01), but not conditioned pain modulation. Greater circulating levels of IL-6 were correlated with enhanced temporal summation of mechanical pain (*r* = 0.304, *P* < 0.01), but the association was no longer significant after adjustment for covariates.

**Conclusion:**

Findings suggest that high levels of some circulating proinflammatory cytokines as well as enhanced pain facilitatory processes together may contribute to the chronic pain experienced by older adults with HIV.

## Abstract 2

Supplemental Digital Content is Available in the Text.

Among 80 people living with HIV and chronic pain, increased age is associated with high levels of circulating proinflammatory cytokines and enhanced pain facilitatory processes.

## 1. Introduction

Current antiretroviral therapy (ART) allows people living with HIV (PLWH) to achieve increased life expectancies that are near normal.^10^ This has led to the aging of the HIV epidemic, with PLWH older than 50 years comprising greater than 40% of Americans living with HIV.^5^ Although the population of PLWH has aged, they have not necessarily aged healthily. People living with HIV have a high burden of other conditions associated with aging including but not limited to heart failure, pulmonary disease, depression, impaired physical function, and chronic pain.^3,18^ These comorbidities could be attributed to risk factors thought of in the HIV-uninfected population and adverse effects of ART, but there is evidence that the HIV virus itself leads to inflammatory processes that are usually associated with normal aging.^3^ The combination of increased age and immune changes caused by the HIV virus has been attributed to CD8^+^ T-cell frequencies in PLWH comparable to HIV-uninfected individuals 20 to 30 years older,^12^ and CD31^+^ CD4 cell frequencies similar to HIV-uninfected individuals 17 to 28 years older.^26^

Chronic pain, defined as pain lasting longer than 3 months or the period of normal tissue healing,^35^ is common and burdensome in both PLWH and in the aging population.^14,20^ The high prevalence of pain in PLWH could be explained by increased pain sensitivity due to both endogenous neural signaling (central and peripheral) and inflammation. A growing body of literature describes how HIV affects peripheral and central nervous system signaling involved in the experience of pain,^7,24^ or endogenous pain modulatory processes.^8^ Increasing age is also associated with a similar pattern, with increased endogenous pain facilitation and decreased pain inhibition with increasing age.^9^ Peripheral markers of inflammation are higher in PLWH than in people without HIV, even in those who achieve virologic suppression.^32^ This is exaggerated further in PLWH with chronic pain. For example, PLWH with chronic widespread pain had significantly elevated levels of interleukin-1β compared to PLWH without chronic pain.^19^ However, it remains unclear whether age may have exerted any effects on these findings. Whether aging is associated with high levels of inflammation in PLWH and chronic pain has not been directly examined to date.

This study had 2 objectives. The first was to examine how age affects endogenous pain modulatory processes in PLWH with chronic pain as assessed by quantitative sensory testing. Our second objective was to examine how age affects serum inflammatory cytokines. Our primary hypothesis was that increasing age is associated with altered endogenous pain modulatory processes (ie, increased facilitation and decreased inhibition). Second, we hypothesized that increasing age is associated with elevated levels of circulating proinflammatory cytokines. Finally, we hypothesized that elevated proinflammatory cytokine levels in PLWH with chronic pain are significantly associated with altered endogenous pain modulatory processes.

## 2. Methods

### 2.1. Participants

People living with HIV and chronic pain were recruited from a large urban, HIV clinic in the southeast United States that provides comprehensive medical, behavioral, and social services to adults with HIV. This clinic provides care to 3457 PLWH, of which 76% are men, 23% are women, and 1% is transgender. Furthermore, 64% identify as Black/African American, 31% as White/Caucasian, and 5% as Hispanic/Others. Study procedures were approved by our institutional review board and conducted in accordance with guidelines for the ethical conduct of research. Written informed consent was obtained from each participant before the study, and they were compensated a total of $200 for their involvement.

### 2.2. Overview of study design

**Figure 1.**
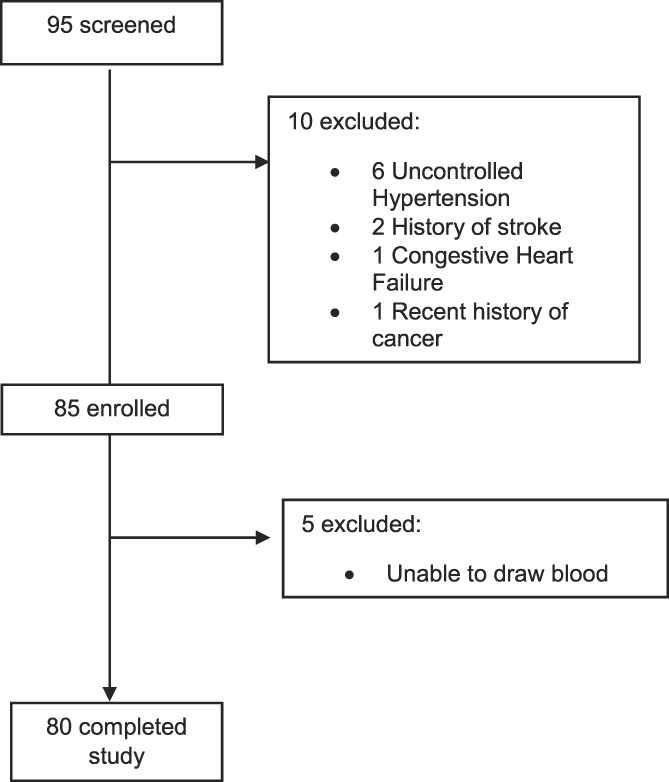
Flow chart depicting matriculation of participants through the study.

A flow diagram depicting matriculation through the study is presented in Figure 1. People living with HIV and chronic pain who were interested in being part of this study were assessed for eligibility during an initial telephone screening.

A review of medical records was completed to validate self-reported personal health histories and determine eligibility. Eligible participants then completed a single study session lasting approximately 2.5 hours. Participants completed resting blood pressure monitoring, blood draws for proinflammatory cytokines (interleukin-6 [IL-6] and tumor necrosis factor-α [TNF-α]), absolute CD-4 helper T-cell count (CD4 count), and HIV viral load (VL), followed by a quantitative sensory testing battery. The quantitative sensory testing battery included temporal summation (TS) of mechanical and heat pain and assessment of conditioned pain modulation (CPM). Finally, all participants completed standardized self-report measures to assess clinical pain severity and pain interference over the past 24 hours.

### 2.3. Eligibility criteria

Eligibility was assessed by telephone screening interviews and medical record review. Inclusion criteria were: (1) HIV-infected; (2) ≥18 years of age; and (3) pain persisting for at least 3 months and resulting in pain on at least half the days in the past 6 months.^33^ Exclusion criteria included: (1) pain-alleviating surgery within the past year; (2) pain intervention (eg, steroid injection) in the past month; (3) evidence of uncontrolled hypertension assessed using sphygmomanometer during study session (ie, resting blood pressure >150/95); (4) history of circulatory disorders or cardiac events; (5) stroke or seizure; (6) metabolic disease; (7) ongoing cancer or cancer treatment; and (8) current pregnancy. Those with uncontrolled hypertension were not eligible for participation in the study due to risk of exacerbating hypertension during the study session. A total of 95 PLWH and chronic pain were screened for study eligibility, 85 were enrolled, and 80 completed the study (Fig. 1).

### 2.4. Medical record review

Prescription data extracted from medical records include ART prescription, and medications that may affect endogenous pain modulation and/or inflammation (eg, antidepressants^2^ or opioid^16^). Furthermore, medical record review was used to confirm participants' self-reported health history provided during telephone screening. Those PLWH and chronic pain whose medical records corroborated their self-reported health history, and who met study inclusion criteria, were deemed eligible for ongoing participation.

### 2.5. Quantitative sensory testing

Participants who reported analgesic medication use including opioids were not asked to withhold these medications before completion of quantitative sensory testing, given that temporary withdrawal from these medications could have affected pain perception. Instead, analgesic medication use was examined and statistically controlled for in data analyses. Methods for quantitative sensory testing that were conducted have previously been detailed by our group.^24^ Briefly, all participants underwent a series of controlled sensory stimulation procedures to assess TS of mechanical and heat pain as a measure of facilitation of nociception, as well as CPM as a measure of inhibition of nociception.^30^ Temporal summation of mechanical pain was always assessed before TS of heat pain, and the TS procedures were always completed before CPM.

#### 2.5.1. Temporal summation of mechanical pain

Temporal summation of mechanical pain was assessed using a nylon monofilament (Touchtest Sensory Evaluator 6.65) at the back on the nondominant hand and the ipsilateral trapezius. Participants provided a verbal 0 to 100 rating of pain after a single contact followed by a rating of greatest pain intensity after a series of 10 contacts at each site conducted twice. Pain ratings at each site were averaged across trials. Temporal summation of mechanical pain was calculated by subtracting pain intensity ratings after the first contact from pain intensity ratings after the 10th contact.

#### 2.5.2. Temporal summation of heat pain

Temporal summation of heat pain was assessed using a Medoc Thermal Sensory Analyzer - II (Medoc, Ltd, Ramat Yishai, Israel) with a 30 × 30-mm diameter thermode using 46°C, 48°C, and 50°C thermal stimuli. Assessment was conducted at midline on the volar surface of the nondominant forearm. A series of 5 heat pulses were presented for each trial, and participants were asked to rate the intensity of the pain produced by each heat pulse on the 0 to 100 numeric rating scale. Temporal summation of heat pain was calculated by subtracting the pain rating from the first heat pulse from the pain rating after the fifth heat pulse.

#### 2.5.3. Conditioned pain modulation

Conditioned pain modulation was tested on the dominant dorsal forearm and ipsilateral trapezius using algometry (test stimulus) and the cold pressor (conditioning stimulus) according to guidelines published by Yarnitsky and colleagues.^37^ A handheld algometer (Medoc, Ltd., AlgoMed) was alternately applied 3 times at each anatomical location, in counterbalanced fashion, to determine participants' baseline pressure pain thresholds (PPTs).^22^ After baseline PPT determination, participants underwent a series of 4 cold-pressor immersions that consisted of placing the nondominant hand, up to the wrist, into a circulating 10°C cold-water bath for 1 minute. Approximately 30 seconds after initiation of each cold-pressor immersion, while the hand was still immersed, the algometer was used to deliver noxious mechanical stimulation to either the dorsal forearm (2 CPM trials) or the ipsilateral trapezius (2 CPM trials); the site order was randomized. Participants again indicated when the increasing pressure stimulation first became painful, representing their conditioned PPTs. There was a 2-minute rest period between each CPM trial. Conditioned pain modulation was calculated by computing a percent change from baseline PPT to conditioning PPT. Percent change scores for each of the 2 CPM trials were calculated according to the following formula: [((conditioned PPT − test PPT)/test PPT) × 100]. This means of computing CPM is consistent with recommendations.^38^ Positive values indicate an inhibitory CPM effect. Negative values are suggestive of pain facilitation (ie, no CPM effect).

### 2.6. Measures

We measured clinical pain severity and interference using the Brief Pain Inventory-Short Form.^31^ This 11-item multidimensional pain scale has demonstrated reliability in patients with neuropathic and musculoskeletal chronic pain conditions. The Coping Strategies Questionnaire Revised measured pain catastrophizing^27^ and the Center for Epidemiological Studies-Depression Scale assessed depression.^25^ We assessed substance use with the Addiction Severity Index^17^ and alcohol use with the Alcohol Use Disorders Identification Test.^29^

#### 2.6.1. Interleukin-6 and tumor necrosis factor-α

The Meso Scale Discovery method was used for the determination of IL-6 and TNF-α proinflammatory cytokine levels. The advantage of Meso Scale Discovery technology is that it allows for multiplexing, or the measurement of more than one biomarker at a time from one sample.

#### 2.6.2. CD4 and HIV viral load

Blood was collected from each participant at the beginning of the study session and sent to the local diagnostics laboratory for quantification of CD4 helper T-cell count and VL. CD4 count was quantified as cells/microliter of blood, whereas VL was quantified as viruses/microliter. Detectable VL was defined as ≥50 copies of virus/mL based on clinical relevance as an HIV treatment endpoint^28^ and to limit alteration of endogenous pain modulation by even small amounts of HIV virus.^7^ All others were considered virologically suppressed (ie, without detectable VL).

### 2.7. Data analyses

All data were analyzed using STATA 15.0 (StataCorp, College Station, TX). We first reviewed data using descriptive summaries and graphical analyses to ensure that values were within appropriate ranges, to check for the presence of outliers and abnormal values, and to verify that the distributions of measures met the assumptions of statistical tests described below. Variables (IL-6) were transformed (log10) if needed in order for variables to meet the assumptions of statistical tests. Descriptive data were assessed using chi-square tests for categorical variables and one-way analysis of variance for continuous variables.

Bivariate analyses were completed to examine whether the quantitative sensory testing measures (change in TS of mechanical pain, change in TS of heat pain, and change in CPM) and markers of inflammation (IL-6 and TNF-α) differed on the variable of interest, age, and potential confounding variables identified a priori based on previous literature and clinical relevance (sex, race/ethnicity, depression, pain severity, catastrophizing, alcohol use, opioid use, HIV VL, and CD4 count). T test or Kruskal–Wallis test was used for categorical variables, and Pearson correlation or Spearman correlation was used for continuous variables. Multivariable analysis was completed for inflammatory markers and quantitative sensory tests that were associated with age at an alpha of 0.05. Covariates that were associated at an alpha of 0.10 or less were included in multivariable analyses.

Multiple linear regression analyses examined the association between age (independent variable) and logIL-6 (dependent variable); age (independent variable) and TS of mechanical pain at the hand (dependent variable); age (independent variable) and TS of mechanical pain at the trapezius (dependent variable); and age (independent variable) and TS of mechanical pain at the hand and trapezius (dependent variable). All tests were 2-tailed and considered statistically significant if *P* < 0.05.

## 3. Results

Participant characteristics are presented in Table 1. Participants were of a mean age of 49 years, ranging from 26 years to 67 years (SD 8), predominantly African American and male. Most participants had an undetectable VL, almost all participants reported being prescribed ART, and most participants reported perfect adherence to their ART in the past month. Among the 19 participants with detectable VL, 11 had a VL greater than 200 copies/mL, and 4 had a VL great than 1000 copies/mL.

**Table 1:** Participant characteristics (n = 80).

	Number, % (unless otherwise specified)
Demographic characteristics	
Age (mean, SD)	49 years (8)
Gender	
Male	53 (66%)
Female	27 (34%)
Race/ethnicity	
Non-Hispanic African American	63 (79%)
Non-Hispanic Caucasian	17 (21%)
Clinical characteristics	
HIV Viral load <50 copies/mL, %	61 (76)
HIV Viral load (copies/mL) (mean, SD)	1008 (6101)
CD4 count (cells/mL) (mean, SD)	645 cells/mm^3^ (334)
Antiretroviral therapy (ART)	
Actively prescribed	79 (99%)
No prescribed	1 (1%)
Opioid use	14 (18%)
AUDIT-C ≥8*	8 (10%)
CES-D ≥16†	54 (68%)
Pain location	
Low back/hips	38 (48%)
Legs/feet	21 (26%)
Arm/hand	5 (6%)
Head	2 (3%)
Multisite	14 (18%)
Pain severity in the past week	
Very mild	7 (9%)
Mild	6 (8%)
Moderate	18 (23%)
Severe	32 (41%)
Very severe	16 (20%)
BPI‡—worst pain (median, IQR), (scale 0–10)	7 (4.5–8)
BPI‡—least pain (median, IQR), (scale 0–10)	6 (3–8)
BPI‡—average pain (median, IQR), (scale 0–10)	7 (5–8)
BPI‡—pain right now (median, IQR), (scale 0–10)	5 (3, 8)
BPI‡—pain interference (median, IQR), (scale 0–10)	4.9 (1.9–6.9)
Cytokines	
IL-6 ng/mL (IQR)	0.9 (0.5–1.7)
TNF-α ng/mL (SD)	3.2 (1.4)
Quantitative sensory testing	
Temporal summation of mechanical pain (mean, SD) (Scale 0–100)	21.3 (22)
Conditioned pain modulation % change—forearm (mean, SD)	16.6 (34)
Conditioned pain modulation % change—trapezius (mean, SD)	13.9 (29.3)
Temporal summation of heat pain (mean, SD) (Scale 0–10)	47.9 (2.3)

* Alcohol Use Disorder Identification Test (AUDIT-C).^24^

† Center for Epidemiological Studies Depression Scale (CES-D).^23^

‡ Brief Pain Inventory.^22^

CES-D, Center for Epidemiological Studies-Depression Scale; IQR, interquartile range; TNF-α, tumor necrosis factor-α.

When identifying the primary location of their pain, almost half of the participants reported pain in the low back and hips. One-quarter of participants reported pain in the legs and feet, and one-fifth reported multisite pain. The majority of participants reported moderate to very severe pain in the past week. Only 14 (18%) reported taking opioids for their pain. More than half of the participants reported moderate or severe depressive symptoms based on the Center for Epidemiological Studies-Depression Scale (Table 1). A CPM effect was demonstrated in 50 (62.5%) participants, and a TS effect was demonstrated in 74 (92.5%) participants.

In bivariate analyses, age in years was significantly associated with logIL-6 (*r* = 0.31, *P* < 0.01) and TS of mechanical pain in the hand and trapezius (*r* = 0.29, *P* < 0.01). Age was not associated with TNF-α, CPM, or TS of heat pain. Sex was significantly associated with logIL-6 (*P* < 0.01). Moderate or severe depressive symptoms were significantly associated with TS of mechanical pain in the hand and trapezius (*P* < 0.05). Circulating levels of logIL-6 were associated with TS of mechanical pain (*r* = 0.30, *P* < 0.01) (Tables 2 and 3; see Supplementary Materials for all bivariate analyses, available at http://links.lww.com/PR9/A81).

**Table 2:** Bivariate associations between covariates and inflammatory markers.

	logIL-6			TNF-α		
	Mean (SD)	*t* test (*df* = 78)	*r*	Mean (SD)	*t* test (*df* = 78)	*r*
Age, y	48.89 (8.17)	—	0.31*	48.89 (8.17)	—	0.09
Race		−0.19	—		−3.51*	—
Black	0.01 (0.39)	—	—	2.9 (1.1)		—
White	0.03 (0.44)	—	—	4.2 (1.9)		—
Gender		−3.47*	—		−0.72	—
Male	−0.08 (0.37)	—	—	3.16 (2.75)	—	—
Female	0.22 (0.39)	—	—	3.40 (2.92)	—	—
Opioid use		0.15	—		0.64	—
No	0.02 (0.41)	—	—	3.28 (1.49)	—	—
Yes	0.003 (0.36)	—	—	3.02 (0.95)	—	—
AUDIT ≥8†		1.48	—		1.12	—
No	0.04 (0.40)	—	—	3.3 (1.45)	—	—
Yes	−0.18 (0.38)	—	—	2.7 (0.84)	—	—
CESD ≥16‡		1.68	—		0.95	—
No	0.13 (0.41)	—	—	3.46 (1.80)	—	—
Yes	−0.03 (0.39)	—	—	3.14 (1.17)	—	—
CD4 count (cells/mm^3^)	645 (334)	—	−0.01	645 (334)	—	−0.18
HIV VL		−1.71	—		−0.89	—
Detectable	−0.12 (0.37)	—	—	2.99 (1.12)	—	—
Undetectable	0.06 (0.41)	—	—	3.32 (1.49)	—	—

* *P* < 0.01.

† Alcohol Use Disorder Identification Test (AUDIT-C).^24^

‡ Center for Epidemiological Studies Depression Scale (CES-D).^23^

*df*, degrees of freedom; IL-6, interleukin-6; *r*, Pearson correlation coefficient; TNF-α, tumor necrosis factor-α; VL, viral load.

**Table 3:** Bivariate associations between covariates and quantitative sensory tests.

	TS of mechanical pain at hand & trapezius scale 0–100)			TS of heat pain* (scale 0–100)			CPM % change at forearm			CPM % change at trapezius		
	Mean (SD)	*T* test	*R*	Mean (SD)	*T* test	*rho*	Mean (SD)	*T* test	*r*	Mean (SD)	*t* test	*r*
Age, y	48.89 (8.17)	—	0.29†	48.89 (8.17)	—	−0.06	48.89 (8.17)	—	0.16	48.89 (8.17)	—	−0.08
Race		1.22	—		−1.60	—		−0.45	—		−3.05†	—
Black	22.90 (22.99)	—		47.78 (2.36)	—		15.74 (34.83)	—		8.92 (24.29)	—	
White	15.54 (18.27)	—		48.74 (1.66)	—		19.97 (31.85)	—		32.18 (38.54)	—	
Gender		−1.4	—		2.4‡	—		1.1	—		0.04	—
Male	18.87 (21.11)	—		48.39 (1.92)	—		19.62 (37.05)	—		13.94 (31.05)	—	
Female	26.19 (23.78)	—		47.13 (2.66)	—		10.79 (26.97)	—		13.69 (25.94)	—	
Opioid use		0.62	—		0.27	—		1.58	—		0.62	—
No	22.04 (22.55)	—		48.01 (2.20)	—		19.39 (35.50)	—		14.80 (31.09)	—	
Yes	18.04 (20.75)	—		47.83 (2.56)	—		3.66 (23.05)	—		9.41 (18.51)	—	
AUDIT ≥8§		1.11	—		−1.03			−0.11			0.05	
No	22.26 (22.59)	—		47.89 (2.29)	—		16.49 (34.00)	—		11.69 (28.27)	—	
Yes	13.09 (16.93)	—		48.75 (1.84)	—		17.9 (37.02)	—		33.31 (32.79)	—	
CESD ≥16‖		2.19‡	—		1.86	—		1.14	—		0.48	—
No	28.96 (28.95)	—		48.64 (1.73)			22.89 (31.85)	—		16.13 (32.15)	—	
Yes	17.67 (17.18)	—		47.66 (2.42)			13.62 (34.96)	—		12.76 (28.01)	—	
CD4 count cells/Ml	645 (334)	—	−0.05	645 (334)	—	0.01	645 (334)	—	−0.09	645 (334)	—	−0.03
HIV VL		0.52	—		0.18	—		1.68	—		0.23	—
Detectable	23.67 (24.74)	—		48.06 (2.03)	—		27.96 (42.02)	—		15.23 (33.59)	—	
Undetectable	20.61 (21.48)	—		47.95 (2.34)	—		13.11 (30.74)	—		13.43 (28.06)	—	
logIL-6 ng/mL	0.02 (0.40)		0.30†	0.02 (0.40)		−0.23‡	0.02 (0.40)	—	−0.02	0.02 (0.40)	—	−0.17
TNF-α ng/mL	3.24 (1.41)		0.20	3.24 (1.41)	—	−0.05	3.24 (1.41)	—	−0.01	3.24 (1.41)	—	0.12

CPM = [((conditioned pressure pain threshold (PPT) − test PPT)/testPPT) × 100].

TS = (pain intensity at 10th contact) − (pain intensity at first contact).

Degree of freedom for all *t* tests is 78, except for TS of heat pain, which is 77.

* 1 missing value.

† <0.01.

‡ <0.05.

§ Alcohol Use Disorder Identification Test (AUDIT-C).^24^

║ Center for Epidemiological Studies Depression Scale (CES-D).^23^

CPM, conditioned pain modulation; IL-6, interleukin-6; PPT, pressure pain threshold; *r*, Pearson correlation coefficient; *rho*, Spearman correlation coefficient; TS, temporal summation; TNF-α- tumor necrosis factor-α; VL, viral load.

In multivariable regression analyses, age was significantly associated with: logIL-6 (*P* < 0.01) when adjusting for race and sex (Table 4); TS of mechanical pain at the trapezius when adjusting for race, sex, and TNF-α (*P* < 0.05) (Table 5); and TS of mechanical pain at the hand and trapezius (*P* < 0.01) when adjusting for race and sex (Table 6). Although logIL-6 was associated with TS of mechanical pain in bivariate analyses, this relationship was no longer significant in multivariable analyses after adjusting for covariates.

**Table 4:** Multivariable linear regression evaluating how age affects log IL-6 (n = 80).

	Beta-coefficient	*P*	95% CI
Age, y	0.01	0.01	0.01 to 0.02
White race*	0.02	0.83	−0.18 to 0.22
Female gender†	0.28	<0.01	0.11 to 0.45

* Compared with black race.

† Compared with male gender.

CI, confidence interval; IL-6, interleukin-6.

**Table 5:** Multivariable linear regression analysis evaluating how age affects temporal summation of mechanical pain at the trapezius.

	Beta-coefficient	*P*	95% CI
Age, y	0.78	0.02	0.14 to 1.42
Race*	−12.21	0.08	−25.73 to 1.32
Gender†	3.42	0.54	−7.53 to 14.37
TNF-α, ng/mL	4.94	0.02	0.96 to 8.92

* Compared with black race.

† Compared with male gender.

CI, confidence interval; TNF-α, tumor necrosis factor.

**Table 6:** Multivariable linear regression evaluating how age affects temporal summation of mechanical pain at the hand and trapezius (n = 80).

	Beta	*P*	95% CI
Age, y	0.74	0.01	0.16 to 1.33
White race*	−7.14	0.22	−18.71 to 4.42
Female gender†	5.98	0.24	−4.09 to 16.04

* Compared with black race.

† Compared with male gender.

CI, confidence interval.

## 4. Discussion

We investigated the relationship between age and circulating levels of inflammatory cytokines with endogenous pain modulatory processes among PLWH and chronic pain. We found that age was significantly associated with increased logIL-6 and endogenous pain facilitation as measured by TS of mechanical pain at the hand and trapezius when adjusting for race and sex, as well as TS of mechanical pain at the trapezius along when adjusting for race, sex, and TNF-α. Aging was not associated with TNF-α, other measures of pain facilitation (TS of heat pain), or pain inhibition (CPM). Markers of inflammation (IL-6 and TNF-α) were not associated with endogenous pain modulatory processes.

As the population of PLWH rapidly ages, management and understanding of chronic comorbidities will increasingly be a major part of HIV care. A clear understanding of the pathogenesis of chronic pain in PLWH will pave the way for future prevention and treatment of chronic pain in this population. HIV infection has been shown to induce production of IL-6 from monocytes and macrophages in PLWH with both suppressed and unsuppressed HIV VL.^4^ Increased circulating inflammatory cytokines (IL-6, IL-2, high sensitivity C-reactive protein, and D-dimer) in PLWH compared with the general population has been linked to many comorbidities that occur at high prevalence among PLWH, including cardiovascular disease, osteoporosis, hypogonadism, and malignancies.^1^

“Inflamm-aging” refers to the chronic, low-grade systemic inflammation that becomes more pervasive as people increase in age, leading to age-associated comorbidities.^6^ IL-6 is associated with aging in PLWH as well as the generally population^15^ and is also linked with chronic pain.^40^ HIV and inflamm-aging processes together likely contribute in a synergistic manner to high levels of circulating proinflammatory cytokines in PLWH.

Inflammation has been shown to promote increased facilitation and decreased inhibition of endogenous pain modulatory processes.^11,36^ We found that age is associated with increased IL-6, a proinflammatory cytokine associated with increased pain sensitivity in PLWH.^34^ However, we did not find any association between aging and TNF-α, and the relationship between inflammation, aging, or other endogenous pain modulatory processes did not reach significance. There is a chance that this relationship is a false positive, and requires repetition in independent and larger data sets.

Our findings show an association between increasing age and endogenous pain modulatory processes. In HIV-uninfected populations, an association between IL-6 and aging has been found in other studies and, in fact, is considered to be the “cytokine of gerontologists” by some.^13^ In our study population, females had significantly higher levels of IL-6 than males. This is also the case in other samples,^21,39^ but has not been as pronounced as in our study. In addition, in other studies, this observation was more pronounced in a narrow range of ages that were postmenopausal.^21^ Our population was somewhat younger than this. We may gain further perspective into why such a pronounced difference in IL-6 was seen in our population by comparing IL-6 in aging PLWH vs those living without HIV. It is possible that the presence of HIV or pain may be contributing to the pronounced effect that we observed in our population.

We found an association between increasing age and TS of mechanical pain. When looking at TS of mechanical pain at the trapezius alone, the association was highly significant when adjusting for race, sex, and TNF-α. However, TNF-α was not associated with TS of mechanical pain when evaluating the hand and trapezius together. In another study performed by our group, TNF-α was associated with TS of mechanical pain at the hand and trapezius.^23^

Our study has limitations. First, we only measured a small set of inflammatory cytokines that are associated with chronic pain and aging. It is also notable that 24% of our sample had detectable VL. The relationships with detectable VL and inflammation and endogenous pain modulation did not meet significance, thus was not included in our final model. It is unknown whether these participants had sustained periods of detectable VL, if their VL was only transiently detectable, and if our findings would have been different if we were able to take these longitudinal factors into account. Our study did not investigate the pathway by which aging led to circulating inflammatory cytokines or endogenous pain modulation.

Future work may evaluate how inflammatory cytokines act as mediators in the relationship between age and endogenous pain modulatory processes. Our study provides the foundation for such work and adds to a small but growing body of research on the role of inflammation in the pathogenesis, treatment, and prevention of chronic pain in PLWH.

## Disclosures

The authors have no conflicts of interest to declare.

## Appendix A. Supplemental digital content

Supplemental digital content associated with this article can be found online at http://links.lww.com/PR9/A81.
